# Supplementary material for: Role and mechanism of NCAPD3 in promoting malignant behaviors in gastric cancer
Source: Front Pharmacol. 2024 Apr 22;15:1341039. doi: 10.3389/fphar.2024.1341039 (PMC11070777; doi:10.3389/fphar.2024.1341039)
Supplement: Supplementary file 11 [file DataSheet2.ZIP › GSEA/Transcipt factor/my_analysis.Gsea.1599461814734/CEBP_C.html]

Details for gene set CEBP\_C[GSEA]

|  || Dataset | filtered\_dataset.sample\_info.cls#WT\_versus\_NCAPD3\_MUT |
| Phenotype | sample\_info.cls#WT\_versus\_NCAPD3\_MUT |
| Upregulated in class | NCAPD3\_MUT |
| GeneSet | CEBP\_C |
| Enrichment Score (ES) | -0.48534524 |
| Normalized Enrichment Score (NES) | -2.3101218 |
| Nominal p-value | 0.0 |
| FDR q-value | 0.017160514 |
| FWER p-Value | 0.077 |
Table: GSEA Results Summary

  

Fig 1: Enrichment plot: CEBP\_C      
 Profile of the Running ES Score & Positions of GeneSet Members on the Rank Ordered List

  

| SYMBOL | TITLE | RANK IN GENE LIST | RANK METRIC SCORE | RUNNING ES | CORE ENRICHMENT || 1 | 324 | APC | 281 | 0.607 | -0.1563 | No |
| 2 | 5295 | PIK3R1 | 467 | 0.493 | -0.2522 | No |
| 3 | 23786 | BCL2L13 | 588 | 0.432 | -0.3059 | No |
| 4 | 6182 | MRPL12 | 729 | 0.359 | -0.3795 | No |
| 5 | 6303 | SAT1 | 850 | -0.233 | -0.4483 | Yes |
| 6 | 6301 | SARS | 856 | -0.259 | -0.4322 | Yes |
| 7 | 1051 | CEBPB | 862 | -0.274 | -0.4150 | Yes |
| 8 | 8848 | TSC22D1 | 899 | -0.324 | -0.4164 | Yes |
| 9 | 80352 | RNF39 | 931 | -0.355 | -0.4118 | Yes |
| 10 | 9572 | NR1D1 | 977 | -0.392 | -0.4145 | Yes |
| 11 | 9180 | OSMR | 981 | -0.394 | -0.3867 | Yes |
| 12 | 9586 | CREB5 | 1009 | -0.409 | -0.3751 | Yes |
| 13 | 117178 | SSX2IP | 1163 | -0.532 | -0.4450 | Yes |
| 14 | 10150 | MBNL2 | 1217 | -0.591 | -0.4383 | Yes |
| 15 | 5187 | PER1 | 1254 | -0.641 | -0.4156 | Yes |
| 16 | 83931 | STK40 | 1281 | -0.669 | -0.3836 | Yes |
| 17 | 5017 | OVOL1 | 1317 | -0.730 | -0.3534 | Yes |
| 18 | 7422 | VEGFA | 1346 | -0.788 | -0.3137 | Yes |
| 19 | 51703 | ACSL5 | 1353 | -0.800 | -0.2573 | Yes |
| 20 | 26298 | EHF | 1378 | -0.919 | -0.2049 | Yes |
| 21 | 5156 | PDGFRA | 1383 | -0.932 | -0.1370 | Yes |
| 22 | 4919 | ROR1 | 1385 | -0.954 | -0.0653 | Yes |
| 23 | 5055 | SERPINB2 | 1397 | -1.088 | 0.0094 | Yes |
Table: GSEA details [plain text format]

  

Fig 2: CEBP\_C      
 Blue-Pink O' Gram in the Space of the Analyzed GeneSet

  

Fig 3: CEBP\_C: Random ES distribution      
 Gene set null distribution of ES for **CEBP\_C**
